# Haplotypes in the *CRP* Gene Associated with Increased BMI and Levels of CRP in Subjects with Type 2 Diabetes or Obesity from Southwestern Mexico

**DOI:** 10.1155/2012/982683

**Published:** 2012-09-25

**Authors:** América Martínez-Calleja, Irma Quiróz-Vargas, Isela Parra-Rojas, José Francisco Muñoz-Valle, Marco A. Leyva-Vázquez, Gloria Fernández-Tilapa, Amalia Vences-Velázquez, Miguel Cruz, Eduardo Salazar-Martínez, Eugenia Flores-Alfaro

**Affiliations:** ^1^Laboratorio de Enfermedades Crónico Degenerativas, Unidad Académica de Ciencias Químico Biológicas, Universidad Autónoma de Guerrero, 39070 Chilpancingo, GRO, Mexico; ^2^Departamento de Biología Molecular y Genómica, Centro Universitario de Ciencias de la Salud, Universidad de Guadalajara, 44350 Guadalajara, JAL, Mexico; ^3^Unidad de Investigación Medica en Bioquímica, Hospital de Especialidades Centro Médico Siglo XXI, Instituto Mexicano del Seguro Social, 06725 México, DF, Mexico; ^4^Centro de Investigación en Salud Poblacional, Instituto Nacional de Salud Pública, 62100 Cuernavaca, MOR, Mexico; ^5^Instituto Mexicano del Seguro Social, 62450 Cuernavaca, MOR, Mexico

## Abstract

*Objective*. We evaluated the association between four polymorphisms in the *CRP* gene with circulating levels of C-reactive protein (CRP), type 2 diabetes (T2D), obesity, and risk score of coronary heart disease. *Methods*. We studied 402 individuals and classified them into four groups: healthy, obese, T2D obese, and T2D without obesity, from Guerrero, Southwestern Mexico. Blood levels of CRP, glucose, cholesterol, triglycerides, and leukocytes were measured. Genotyping was performed by PCR/RFLP, and the risk score for coronary heart disease was determined by the Framingham's methodology. *Results*. The TT genotype of SNP rs1130864 was associated with increased body mass index and T2D patients with obesity. We found that the haplotype 2 (TGAG) was associated with increased levels of CRP (*β* = 0.3; 95%CI: 0.1, 0.5; *P* = 0.005) and haplotype 7 (TGGG) with higher body mass index (BMI) (*β* = 0.2; 95%CI: 0.1, 0.3; *P* < 0.001). The risk score for coronary heart disease was associated with increased levels of CRP, but not with any polymorphism or haplotype. *Conclusions*. The association between the TT genotype of SNP rs1130864 with obesity and the haplotype 7 with BMI may explain how obesity and genetic predisposition increase the risk of diseases such as T2D in the population of Southwestern Mexico.

## 1. Introduction

It has been proposed that the C-reactive protein (CRP) has clinical utility as a biomarker in predicting risk of cardiovascular events [1] and a predictive value in the progression of type 2 diabetes (T2D) [2]. In obesity, there is an increase in the risk of coronary heart disease (CHD) by constant and progressive vascular damage, generated by a chronic low-grade inflammatory state [3]. It has been determined that adipocytes produce one-third of interleukin-6 (IL-6), the main stimulus for hepatic CRP production. Overweight or obese subjects have higher levels of this protein [4], which promotes the atherosclerotic process by increasing the expression of adhesion molecules (ICAM-1, VCAM-1), E-selectin, and the monocyte chemotactic protein-1 (MCP-1) in the endothelium [5]. CRP is also involved in decreased expression and mRNA half-life of endothelial nitric oxide synthase and activation of nuclear factor kappa B (NF*κ*B) that is involved in the transcription of numerous genes related with the inflammatory process [5]. It has also been involved in the modulation of angiogenesis and atherosclerotic, plaque instability by activating the signaling pathway of phosphatidylinositol-3 kinase (PI3 K) and extracellular signal-regulated kinases 1 and 2 (ERK 1/2) [6].

 Obesity, age, gender, and diabetes are important factors that influence variation in blood levels of CRP. Also, there is a strong evidence of the genetic component in the variation of this protein, with heritability estimates of 35–40% [7]. Several studies have reported an association between single-nucleotide polymorphisms (SNPs) in the *CRP* gene with variation in blood levels of CRP, or with CHD, diabetes, microangiopathic stroke, insulin resistance, metabolic syndrome, or hypertension [8–10]. In particular, polymorphisms in the *CRP *gene on chromosome 1 have consistently been associated with basal CRP levels in both men and women [9, 11] and with varying degrees of risk in the development of CHD [8], while polymorphisms within the *CRP *promoter have been associated with differential transcription of *CRP *in clinically stable patients [12]. However, other studies have not been able to identify an association between *CRP* genotypes and the risk of CHD [13, 14]. 

Several studies report an association between single-nucleotide polymorphisms (SNPs) in the CRP gene with variation in blood levels of CRP with cardiovascular disease (CVD) and other diseases such as diabetes, microangiopathic stroke, insulin resistance, metabolic syndrome, and hypertension [12]. On the other hand, in the Mexican population, few studies have shown a relationship between *CRP* gene variants with blood levels of the protein in obesity, diabetes, and cardiovascular risk. Our research was conducted to evaluate the association between genotype and haplotype frequencies of SNPs rs1130864, rs1205, rs2794521, and rs3093062 in the *CRP* gene with serum levels of CRP, in healthy, obese, T2D obese, and T2D without obesity, or with the risk score of CHD. Currently, Mexico has one of the highest obesity rates in the world; one-third of children in Mexico are now classified as either overweight or obese [15].

## 2. Subjects and Methods

### 2.1. Previously Diagnosed

#### 2.1.1. Subjects

A total of 402 individuals, 30 years or older, male and female, normotensive, genetically unrelated, and with ancestry in the state of Guerrero, Mexico, were studied. Patients from the ISSSTE Hospital Clinic and Health Center of Chilpancingo, Guerrero were invited to participate in the study. Patients were classified into four groups: 130 healthy individuals not overweight (BMI < 25 kg/m^2^), 93 previously diagnosed with T2D without obesity (BMI < 25 kg/m^2^), 106 obese subjects (BMI > 30 kg/m^2^), and 73 previously diagnosed with T2D and obesity (BMI > 30 kg/m^2^). Smokers, alcohol consumers, those with a diagnosis of CHD, respiratory tract infection, digestive or urinary tract in the last 15 days, or treated with anti-inflammatory drugs, or pregnant women were excluded. Individuals that accepted to participate signed the informed consent form. The protocol was approved by the Ethics Committee of the University of Guerrero. Each participant answered a questionnaire to obtain sociodemographic, family history, and history of diseases data. Additionally, weight (kg), height (m), waist circumference (cm), and blood pressure (mmHg) were measured. Fasting blood samples were obtained from the study subjects.

#### 2.1.2. Laboratory Measurements

The serum levels of glucose and triglycerides were obtained using routine biochemical analysis. High-sensitivity CRP levels were measured by immunenephelometry by the automated system BN-100 (Dade Behring, Germany). The intra- and interassay coefficients of variation of CRP were *<*4.2% and *<*5.5%, respectively, and the analytical sensitivity was 0.175 mg/L. The total count of leukocytes was measured in the hematologic system ADVIA-60 (Bayer, USA) the percentage of glycosylated hemoglobin (HbA1c) was performed by the technique of agglutination inhibition (DCA-2000, Bayer, USA).

#### 2.1.3. Genotyping

DNA was extracted from peripheral blood leukocytes using Miller's technique [16]. Genotyping of the *CRP *SNPs +1444 C > T (rs1130864), +1846 G > A (rs1205), −717 A > G (rs2794521), and −409 G > A (rs3093062) was done via PCR-restriction fragment length polymorphism (RFLP) as previously described [8, 9, 17, 18]. The digested fragments were then separated by electrophoresis in 4% agarose gels, followed by ethidium bromide staining and visualized under ultraviolet light. To improve genotyping accuracy, samples with known genotypes were used in each batch as positive controls to evaluate the completeness of PCR product. Details of genotyping method are shown in Table 1.

#### 2.1.4. Statistical Analyses

Quantitative variables were summarized as medians and 25–75 percentiles or geometric means and standard error, and in frequencies for qualitative variables. CHD risk score was stratified by gender, age, blood pressure, cholesterol, triglycerides, and diabetes, a methodology proposed by Wilson et al. [19]. To compare the different factors or SNPs with the study groups, Kruskal-Wallis or chi-square (*χ* ^2^) tests were used. The relationship between CRP and the different variables was determined using the Spearman correlation coefficient. The effect of genotypes and haplotypes of SNPs in the *CRP* gene on CRP levels and BMI was assessed by linear regression models after logarithmic transformation of CRP and BMI. Furthermore, we evaluated multinomial logistic regression models to determine association between SNPs and study groups. Statistical analysis was performed using STATA software (v.11.1). Uncorrected (*P* < 0.05) or corrected (*P* < 0.0129) *P* values for the analysis of SNPs based on multiple comparisons correction method proposed by Cheverud [20] were considered statistically significant. Haplotypes were constructed using the genetic data analysis program SNPstats (http://bioinfo.iconcologia.net/SNPstats).

## 3. Results

The clinical and demographic characteristics of the study groups are summarized in Table 2. Subjects with T2D not obese, obese, and T2D obese individuals showed the highest values in different cardiovascular risk factors compared with healthy individuals. Serum CRP was higher in obese individuals (3.6 mg/L) and T2D obese (4.4 mg/L) compared with not obese. Meanwhile, the CHD risk score was higher in individuals with T2D without obesity.

In order to evaluate the risk of CHD, the CRP levels thresholds used were low risk (<1.0 mg/L), average risk (1.0 to 3.0 mg/L) and high risk (>3.0 mg/L), based on the criteria of the Centers for Disease Control and the American Heart Association [21]. We found in high risk of CHD 45% of all individuals, higher in T2D obese (74%) and obese (66%) compared with healthy subjects (21.5%), and only 25.1% of all subjects were found in low-risk stratum (Table 2). The CRP blood concentration was significantly correlated with BMI (*P* < 0.001), systolic blood pressure (*P* = 0.022), glucose (*P* < 0.001), triglycerides (*P* = 0.022), total leukocyte count (*P* < 0.001), and the CHD risk score (*P* = 0.038). On the other hand, the increase in the risk score had a significant effect on increasing levels of CRP (*P* = 0.002) adjusted for BMI (data not shown).

 The frequency of individuals carrying the TT genotype of SNP rs1130864 was 19.4%, 25.9% for AA of rs1205, 2.2% for GG genotype of rs2794521, and for AA genotype of SNP rs3093062 was not observed in the studied population. The polymorphisms in the healthy group followed the Hardy-Weinberg equilibrium (*P* > 0.05). The SNP rs1130864 TT genotype was more prevalent in individuals with T2D and obese (*P* = 0.029) (Table 3).

 The SNP rs1130864 TT genotype had a marginal effect on increasing levels of CRP and BMI, while the AA genotype of SNP rs1205 influenced the decrease in CRP (*P* = 0.002) by codominant models of inheritance. We also found a significant trend in the increase of the average concentration of CRP between the genotypes of the SNPs rs1130864 and rs3093062 and a decreasing trend in theconcentration of CRP with the SNP rs1205 (Table 4). Additionally, we found an association between the TT genotype of polymorphism rs1130864 with T2D not-obese group (OR = 4.7, 95% CI; 1.7–12.9, *P* = 0.003), the obese group (OR = 4.4, 95% CI; 1.9–10.2, *P* = 0.001), and the group of T2D obese (OR = 5.6, 95% CI; 2.0–15.5, *P* = 0.001), compared with the healthy group, in models adjusted for CHD risk score and region of origin, with a significance level (*α*) of 0.0129. We did not find a significant association between the SNPs with CHD risk score (data not shown).

 Haplotype 2 (TGAG) showed a significant association with increased serum levels of CRP and marginally with haplotype 7 (TGGG), compared with the more frequent haplotype (CAAG), while with the increase in BMI it was associated only with haplotype 7 (Table 5).

## 4. Discussion

It has been shown that elevated serum CRP is a risk factor for CHD, and there is a relationship between increased serum levels of CRP with various CHD risk factors, particularly diabetes and hypertension [22, 23]. On the other hand, it has been reported that several polymorphisms in the *CRP* gene are associated with variation in the concentration of this protein, which is increased with the TT and GG genotypes of the SNPs rs30864 and rs2794521, respectively, and decreased with the AA genotype of rs1205. Also, several alterations have been associated with metabolic syndrome, hypertension, insulin resistance, obesity, and cardiovascular disease, among others [24, 25].

Our results are consistent with those reported in different populations of the world where polymorphisms in CRP gene are related with changes in serum levels of CRP [11, 24], indicating that their effect on the protein is independent of race/ethnicity. Furthermore, the results confirm the effect of obesity and diabetes on circulating levels of CRP described by other authors. Indicating that in individuals with a low*-*grade chronic systemic inflammation, this environment favors the development of atherosclerosis mediated by a process of endothelial dysfunction [2, 4]. We also found that the increase in CHD risk score had a significant effect on increasing levels of CRP; however, this score was not associated with any of the polymorphisms studied. The novelty of the results presented here is the association between the TT genotype of SNP rs1130864 with obesity and T2D and the haplotypes 2 (TGAG) and 7 (TGGG) with increase of CRP and BMI, respectively.

The regulatory effect of IL-6 on CRP gene transcription is a process involving several hepatic transcription factors (C/EBP, HNF, and NF*κ*B). The promoter region of the CRP gene also has binding sites for STAT3 and Rel proteins, where the interaction between these factors gives more stability to the binding C/EBP to DNA and results in maximum gene induction [26]. Furthermore, the 3′UTR region is associated with increased mRNA stability in T allele carriers of SNP rs1130864 and for the G allele of SNP rs1205 and therefore increased expression of CRP [17].

Two regions in chromosomes 1 and 6 with T2D (1q21–q25 and 6q21–q23) have been related by genome scan studies [27, 28], where the CRP gene is located on the 1q21–q23 region (http://www.genenames.org/data/hgnc_data.php?hgnc_id=2367). Furthermore, an association has been reported of several SNPs in the CRP gene with T2D [10, 23, 29], suggesting that this may be a candidate gene involved in the development of T2D, a frequent disease in individuals with obesity. We found association between individuals carrying the TT genotype of SNP rs1130864 with obesity or with increased BMI and T2D. This strengthens the hypothesis that CRP may contribute to the development of T2D [23, 29]. Eiriksdottir et al. [30] reported a significant interaction between BMI and waist circumference with the AA genotype of SNP rs1205 on CRP levels, indicating that this effect could explain why obesity promotes a more stable mRNA through the G allele, and the strong linkage disequilibrium has been found in different blocks of the CRP gene, as well as in the 3′UTR region in the block formed by the SNPs rs1130864 and rs1205. Casas et al. [31] found significant differences in mean BMI among individuals with TT genotype compared with C allele carriers, meanwhile, Teng et al. [32] found association between the interaction of obesity with SNPs rs2794521 and rs1800947 (*P* = 0.034 and *P* = 0.020, resp.), with increased serum levels of CRP. The SNP rs1130864 TT genotype has been consistently associated with increased serum levels of CRP [17, 24] and has been associated with cardiovascular events [9, 33].

Several studies have made efforts to determine the effect of haplotypes on certain diseases or phenotypic traits. Different haplotypes in the *CRP* gene have been associated with changes in serum levels of CRP [11, 24], with microangiopathic damage, or T2D [23]. We found that haplotypes 2 and 7 were associated with an increase in levels of CRP and IMC, respectively. 

## 5. Conclusion

In summary, our results in the population from Southwestern Mexico indicate that the variation in the CRP gene is associated not only with basal levels of CRP, but also with other disorders such as obesity and T2D, and an association was found with genotype TT of rs1130864 and haplotype 7 (TGGG). Genetic effects on phenotypic traits or diseases are generally small; however, it is possible that a set of polymorphisms together with environmental factors contribute to the development of disease, and obesity to the development of several chronic degenerative diseases modulates the increased risk of these diseases through the genes. 

## Figures and Tables

**Table 1:** PCR primers, products, and restriction enzymes for the genotyping of *CRP* polymorphisms.

SNP	Forward and reverse primers	PCR product (bp)	Restriction enzyme	Fragments (bp)			Reference
rs1130864	5′-AGCTCGTTAACTATGCTGGGGCA-3′ 5′-CTTCTCAGCTCTTGCCTTATGAGT-3′	181	*Hp* *yC* *H*_4_ *III *	CC 181	CT 181, 156, 25	TT 156, 25	[9]

rs1205	5′-GGAGTGAGACATCTTCTTG-3′ 5′-CTTATAGACCTGGGCAGT-3′	227	*Hp* *yC* *H*_4_ *III *	GG 130 y 97	GA 227, 130, 97	AA 227	[17]

rs2794521	5′-GCCGTCATTTAGTGCCAAC-3′ 5′-ATGCTCCTCCCAGAGCCATGG-3′	376	*Bstul*	AA 376	AG 376, 199, 177	GG 199, 177	[18]

rs3093062	5′-TTTGGGCTAAGTAGGTGTTG-3′ 5′-AGGGCTCCACTTTGGCTATC-3′	116	*Apal I*	GG 38, 78	GA 116	AA 38, 78, 116	[8]

**Table 2:** Clinical and demographic characteristics of patients in the study groups.

Characteristics	Healthy	T2D without obesity	Obese	T2D with obesity	*P* value
	*n* = 130	*n* = 93	*n* = 106	*n* = 73	
Age (years)	40 (35–47)	56 (46–67)	43 (38–49)	48 (43–59)	<0.001^a^
Gender, *n* (%)					
Male	33 (25.4)	30 (32.3)	23 (21.7)	21 (28.8)	0.376^b^
Female	97 (74.6)	63 (67.7)	83 (78.3)	52 (71.2)	
Body mass index (kg/m^2^)	23.6 (22.2–24.6)	24.0 (22.4–24.7)	33.1 (30.8–35.3)	32.0 (30.3–34.3)	<0.001^a^
Waist (cm)	82 (76–87)	86 (82–92)	103 (97–108)	103 (98–108)	<0.001^a^
Abdominal obesity, *n* (%)	14 (10.8)	18 (19.4)	97 (91.5)	66 (90.4)	<0.001^b^
Systolic blood pressure (mmHg)	107 (95–115)	118 (110–129)	109 (101–123)	112 (103–120)	<0.001^a^
Diastolic blood pressure (mmHg)	69 (63–76)	71 (66–79)	72 (67–79)	73 (67–77)	0.006^a^
Glucose (mg/dL)	84 (77–89)	174 (120–243)	89 (84–96)	151 (120–222)	<0.001^a^
Cholesterol (mg/dL)	197 (167–220)	185 (165–206)	185 (156–218)	184 (161–215)	0.320^a^
Triglycerides (mg/dL)	141 (99–187)	181 (126–250)	148 (107–187)	191 (142–249)	<0.001^a^
Leukocytes (10^3^/*μ*L)	5.8 (5.1–6.2)	6.7 (5.7–6.9)	6.6 (5.7–7.5)	6.4 (5.7–7.0)	<0.001^a^
hsCRP (mg/L)*	1.2 (0.17)	1.7 (0.33)	3.6 (0.40)	4.4 (0.45)	<0.001^a^
<1	58 (44.6)	27 (29.0)	11 (10.4)	5 (6.8)	<0.001^b^
1–3	44 (33.9)	36 (38.7)	25 (23.6)	14 (19.2)	
>3	28 (21.5)	30 (32.3)	70 (66.0)	54 (74.0)	
CHD risk score	−0.5 (−6, 3)	9 (5, 12)	1 (−5, 4)	6 (4, 9)	<0.001^a^

The data indicate median (p25–p75), *n* (%) *geometric mean (standard error).

^a^Kruskall-Wallis's test; ^b^ *χ* ^2^ test.

hsCRP: C-reactive protein high sensitivity. CHD: coronary heart disease.

**Table 3:** Genotypic and allelic frequencies of polymorphisms in the CRP gene by study groups.

SNP	All	Healthy	T2D without obesity	Obesity	T2D with obesity	*P* value
	*n* (%)	*n* (%)	*n* (%)	*n* (%)	*n* (%)	
rs1130864						
CC	108 (26.9)	47 (36.2)	25 (26.9)	20 (18.9)	16 (21.9)	0.029^a^
CT	216 (53.7)	68 (52.3)	47 (50.5)	61 (57.5)	40 (54.8)	
TT	78 (19.4)	15 (11.5)	21 (22.6)	25 (23.6)	17 (23.3)	
C	432 (53.7)	162 (62.3)	97 (52.1)	101 (47.6)	72 (49.3)	
T	372 (46.3)	98 (37.7)	89 (47.9)	111 (52.4)	74 (50.7)	
HWE: *χ* ^2^ (*P*)		1.7 (0.20)				

rs1205						
GG	127 (31.6)	37 (28.5)	31 (33.3)	35 (33.0)	24 (32.9)	0.866^a^
GA	171 (42.5)	55 (42.3)	37 (39.8)	45 (42.5)	34 (46.6)	
AA	104 (25.9)	38 (29.2)	25 (26.9)	26 (24.5)	15 (20.6)	
G	425 (52.9)	129 (49.6)	99 (53.2)	115 (54.3)	82 (56.2)	
A	379 (47.1)	131 (50.4)	87 (46.8)	97 (45.8)	64 (43.8)	
HWE: *χ* ^2^ (*P*)		3.0 (0.08)				

rs2794521						
AA	314 (78.1)	111 (85.4)	68 (73.1)	81 (76.4)	54 (74.0)	0.281^b^
AG	79 (19.7)	17 (13.1)	22 (23.7)	23 (21.7)	17 (23.3)	
GG	9 (2.2)	2 (1.5)	3 (3.2)	2 (1.9)	2 (2.7)	
A	707 (87.9)	239 (91.9)	158 (85.0)	185 (87.3)	125 (85.6)	
G	97 (12.1)	21 (8.1)	28 (15.0)	27 (12.7)	21 (14.4)	
HWE: *χ* ^2^ (*P*)		1.9 (0.174)				

rs3093062						
GG	378 (94.0)	127 (97.7)	86 (92.5)	98 (92.5)	67 (91.8)	0.137^b^
GA	24 (6.0)	3 (2.3)	7 (7.5)	8 (7.5)	6 (8.2)	
G	780 (97.0)	257 (98.9)	179 (96.2)	204 (96.2)	140 (95.9)	
A	24 (3.0)	3 (1.1)	7 (3.8)	8 (3.8)	6 (4.1)	
HWE: *χ* ^2^ (*P*)		0.02 (0.894)				

The data indicate *n* (%). HWE: Hardy-Weinberg equilibrium.

^a^ *χ* ^2^ test; ^b^Fisher's exact test.

**Table 4:** Effect of the SNPs on hsCRP levels or BMI.

SNP	CRP (mg/L)	Log CRP (mg/L)		BMI (kg/m^2^)	Log BMI (kg/m^2^)	
	GM (95%CI)	*β* (95%CI)^a^	*P* value	GM (95%CI)	*β* (95%CI)^b^	*P* value^c^
rs1130864						
CC	1.7 (1.4–2.1)	Reference		26.2 (25.3–27.1)	Reference	
CT	2.3 (2.0–2.6)	0.2 (0.05, 0.4)	0.128	27.4 (26.7–28.1)	0.04 (−0.02, 0.1)	0.065
TT	2.9 (2.2–3.9)	0.4 (0.1, 0.7)	0.015	28.2 (26.8–29.7)	0.1 (0.01, 0.1)	0.016
*P* trend	0.001			0.012		
CT + TT	2.4 (2.1–2.8)	0.2 (0.1, 0.6)	0.045	27.6 (27.0–28.3)	0.05 (0.01, 0.1)	0.024

rs1205						
GG	2.5 (2.0–3.0)	Reference		27.5 (27.0–29.4)	Reference	
GA	2.6 (2.2–3.0)	0.1 (−0.2, 0.3)	0.423	27.4 (26.6–28.2)	−0.01 (−0.1, 0.04)	0.871
AA	1.5 (1.2–1.9)	−0.4 (−0.7, −0.2)	0.002	26.6 (25.7–27.6)	−0.03 (−0.1, 0.02)	0.197
*P* trend	0.001			0.220		
GA + AA	2.1 (1.8–2.4)	−0.1 (−0.3, −0.1)	0.334	27.1 (26.5–27.7)	−0.02 (−0.1, 0.02)	0.477

rs2794521						
AA	2.1 (1.9–2.4)	Reference		27.0 (26.4–27.6)	Reference	
AG	2.7 (2.1–3.4)	0.1 (−0.1, 0.4)	0.317	28.1 (26.8–29.4)	0.04 (−0.01, 0.1)	0.105
GG	2.1 (0.7–6.0)	−0.1 (−0.8, 0.4)	0.747	28.2 (24.2–32.9)	0.04 (−0.1, 0.2)	0.501
*P* trend	0.164			0.109		
AG + GG	2.6 (2.1–3.3)	0.1 (−0.1, 0.3)	0.398	28.1 (26.9–29.4)	0.04 (−0.01, 0.08)	0.087

rs3093062						
GG	2.1 (1.9–2.4)	Reference		27.1 (26.6–27.7)	Reference	
GA	3.7 (2.4–5.9)	0.3 (−0.1, 0.8)	0.108	29.4 (26.6–32.3)	0.1 (−0.01, 0.2)	0.060
*P* trend	0.020			0.060		

^a^Models adjusted by risk score of coronary heart disease, body mass index, and place of origin.

^b^Models adjusted by risk score of coronary heart disease and place of origin. GM: geometric mean.

^c^Uncorrected *P* values.

**Table 5:** Association of haplotypes in CRP gene with circulating levels of CRP and body mass index.

Haplotype	SNP					Log CRP (mg/L)		Log BMI (kg/m^2^)	
	rs1130864	rs1205	rs2794521	rs3093062	Frequency	*β* (95%CI)^a^	*P* value	*β* (95%CI)^b^	*P* value
1	C	A	A	G	0.2934	Ref		Ref	
2	T	G	A	G	0.2818	0.3 (0.1, 0.5)	0.005	0.04 (0, 0.1)	0.059
3	C	G	A	G	0.1523	0.2 (−0.01, 0.5)	0.057	0.01 (−0.04, 0.06)	0.57
4	T	A	A	G	0.127	0.2 (−0.1, 0.4)	0.27	0.04 (−0.02, 0.1)	0.17
5	C	G	G	G	0.0403	0.1 (−0.4, 0.5)	0.72	−0.03 (−0.1, 0.1)	0.46
6	C	A	G	G	0.0389	0.1 (−0.3, 0.6)	0.59	0.07 (−0.03, 0.2)	0.16
7	T	G	G	G	0.0291	0.7 (0.2, 1.2)	0.01	0.2 (0.1, 0.3)	<0.001
8	C	G	A	A	0.0101	0.7 (−0.04–1.5)	0.063	0.2 (0, 0.3)	0.049
Rare					0.027	0.6 (0.1, 1.1)	0.015	0.1 (−0.1, 0.2)	0.29

^a^Models adjusted by risk score of coronary heart disease, body mass index, and place of origin.

^b^Models adjusted by risk score of coronary heart disease and place of origin.

^c^Uncorrected *P* values.

## References

[1] Musaad S, Haynes EN (2007). Biomarkers of obesity and subsequent cardiovascular events. *Epidemiologic Reviews*.

[2] Kahn SE, Zinman B, Haffner SM (2006). Obesity is a major determinant of the association of C-reactive protein levels and the metabolic syndrome in type 2 diabetes. *Diabetes*.

[3] Pradhan AD, Manson JE, Rifai N, Buring JE, Ridker PM (2001). C-reactive protein, interleukin 6, and risk of developing type 2 diabetes mellitus. *Journal of the American Medical Association*.

[4] Lau DCW, Dhillon B, Yan H, Szmitko PE, Verma S (2005). Adipokines: molecular links between obesity and atheroslcerosis. *American Journal of Physiology—Heart and Circulatory Physiology*.

[5] Paffen E, deMaat MPM (2006). C-reactive protein in atherosclerosis: a causal factor?. *Cardiovascular Research*.

[6] Bello G, Cailotto F, Hanriot D (2008). C-reactive protein (CRP) increases VEGF-A expression in monocytic cells via a PI3-kinase and ERK 1/2 signaling dependent pathway. *Atherosclerosis*.

[7] Pankow JS, Folsom AR, Cushman M (2001). Familial and genetic determinants of systemic markers of inflammation: the NHLBI family heart study. *Atherosclerosis*.

[8] Szalai AJ, Wu J, Lange EM (2005). Single-nucleotide polymorphisms in the C-reactive protein (CRP) gene promoter that affect transcription factor binding, alter transcriptional activity, and associate with differences in baseline serum CRP level. *Journal of Molecular Medicine*.

[9] Brull DJ, Serrano N, Zito F (2003). Human CRP gene polymorphism influences CRP levels: implications for the prediction and pathogenesis of coronary heart disease. *Arteriosclerosis, Thrombosis, and Vascular Biology*.

[10] Wolford JK, Gruber JD, Ossowski VM (2003). A C-reactive protein promoter polymorphism is associated with type 2 diabetes mellitus in Pima Indians. *Molecular Genetics and Metabolism*.

[11] Miller DT, Zee RYL, Suk Danik J (2005). Association of common CRP gene variants with CRP levels and cardiovascular events. *Annals of Human Genetics*.

[12] Carlson CS, Aldred SF, Lee PK (2005). Polymorphisms within the C-reactive protein (CRP) promoter region are associated with plasma CRP levels. *American Journal of Human Genetics*.

[13] Zacho J, Tybjærg-Hansen A, Jensen JS, Grande P, Sillesen H, Nordestgaard BG (2008). Genetically elevated C-reactive protein and ischemic vascular disease. *New England Journal of Medicine*.

[14] Lawlor DA, Harbord RM, Timpson NJ (2008). The association of C-reactive protein and CRP genotype with coronary heart disease: findings from five studies with 4,610 cases amongst 18,637 participants. *PLoS One*.

[15] http://geo-mexico.com/?wpsc-product=geo-mexico-the-geography-and-dynamics-of-modern-mexico.

[16] Miller SA, Dykes DD, Polesky HF (1988). A simple salting out procedure for extracting DNA from human nucleated cells. *Nucleic Acids Research*.

[17] Russell AI, Cunninghame-Graham DS, Shepherd C (2004). Polymorphism at the C-reactive protein locus influences gene expression and predisposes to systemic lupus erythematosus. *Human Molecular Genetics*.

[18] Chen J, Zhao J, Huang J, Su S, Qiang B, Gu D (2005). -717A>G polymorphism of human C-reactive protein gene associated with coronary heart disease in ethnic Han Chinese: the Beijing atherosclerosis study. *Journal of Molecular Medicine*.

[19] Wilson PWF, D’Agostino RB, Levy D, Belanger AM, Silbershatz H, Kannel WB (1998). Prediction of coronary heart disease using risk factor categories. *Circulation*.

[20] Cheverud JM (2001). A simple correction for multiple comparisons in interval mapping genome scans. *Heredity*.

[21] Sabatine MS, Morrow DA, Jablonski KA (2007). Prognostic significance of the Centers for Disease Control/American Heart Association high-sensitivity C-reactive protein cut points for cardiovascular and other outcomes in patients with stable coronary artery disease. *Circulation*.

[22] Lange LA, Carlson CS, Hindorff LA (2006). Association of polymorphisms in the CRP gene with circulating C-reactive protein levels and cardiovascular events. *Journal of the American Medical Association*.

[23] Dehghan A, Kardys I, de Maat MPM (2007). Genetic variation, C-reactive protein levels, and incidence of diabetes. *Diabetes*.

[24] Kathiresan S, Larson MG, Vasan RS (2006). Contribution of clinical correlates and 13 C-reactive protein gene polymorphisms to interindividual variability in serum C-reactive protein level. *Circulation*.

[25] Han TS, Sattar N, Williams K, Gonzalez-Villalpando C, Lean MEJ, Haffner SM (2002). Prospective study of C-reactive protein in relation to the development of diabetes and metabolic syndrome in the Mexico City diabetes study. *Diabetes Care*.

[26] Kushner I, Jiang SL, Zhang D, Lozanski G, Samols D (1995). Do post-transcriptional mechanisms participate in induction of C-reactive protein and serum amyloid A by IL-6 and IL-1?. *Annals of the New York Academy of Sciences*.

[27] Xiang K, Wang Y, Zheng T (2004). Genome-wide search for type 2 diabetes/impaired glucose homeostasis susceptibility genes in the Chinese: significant linkage to chromosome 6q21-q23 and chromosome 1q21-q24. *Diabetes*.

[28] Ng MCY, So WY, Cox NJ (2004). Genome-wide scan for type 2 diabetes loci in Hong Kong Chinese and confirmation of a susceptibility locus on chromosome 1q21-q25. *Diabetes*.

[29] Zee RYL, Germer S, Thomas A (2008). C-reactive protein gene variation and type 2 diabetes mellitus: a case-control study. *Atherosclerosis*.

[30] Eiriksdottir G, Smith AV, Aspelund T (2009). The interaction of adiposity with the CRP gene affects CRP levels: Age, Gene/Environment Susceptibilty-Reykjavik Study. *International Journal of Obesity*.

[31] Casas JP, Shah T, Cooper J (2006). Insight into the nature of the CRP-coronary event association using Mendelian randomization. *International Journal of Epidemiology*.

[32] Teng MS, Hsu LA, Wu S, Chang HH, Chou HH, Ko YL (2009). Association between C-reactive protein gene haplotypes and C-reactive protein levels in Taiwanese: interaction with obesity. *Atherosclerosis*.

[33] Kuhlenbaeumer G, Huge A, Berger K (2010). Genetic variants in the C-reactive protein gene are associated with microangiopathic ischemic stroke. *Cerebrovascular Diseases*.

